# P-1186. Pharmacodynamic Assessment of Combination of Cefiderocol with Xeruborbactam in Murine Thigh Infection Model

**DOI:** 10.1093/ofid/ofaf695.1379

**Published:** 2026-01-11

**Authors:** Eri Mizusawa, Masaaki Izawa, Sachi Kanazawa, Takafumi Hara, David Griffith, Yoshinori Yamano

**Affiliations:** Shionogi & Co., Ltd., Toyonaka, Osaka, Japan; Shionogi & Co., Ltd., Toyonaka, Osaka, Japan; Shionogi & Co., Ltd., Toyonaka, Osaka, Japan; Shionogi & Co., Ltd., Toyonaka, Osaka, Japan; Qpex Biopharma, San Diego, California; Shionogi & Co., Ltd., Toyonaka, Osaka, Japan

## Abstract

**Background:**

Cefiderocol (FDC) is a siderophore-conjugated cephalosporin with potent activity against gram-negative bacteria including carbapenem-resistant strains. Higher minimum inhibitory concentrations (MICs) to FDC have been observed with isolates producing NDM- or PER/VEB-type β-lactamases together with other factors like iron transporters and PBP3 mutations. Xeruborbactam (XER) is a novel β-lactamase inhibitor that inhibits serine- and metallo-type β-lactamases. In this study, we evaluated the impact of XER on the efficacy of FDC in a murine thigh infection model.

**Figure:**
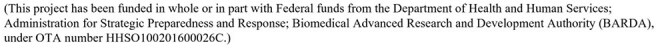

**Table.**
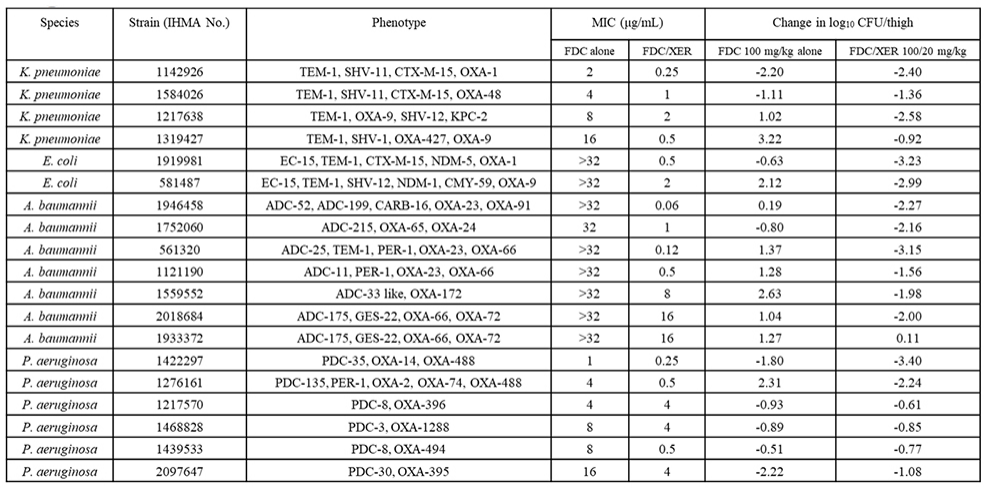
Efficacy of FDC alone and in combination with XER against 19 strains in a murine thigh infection model MICs of FDC were determined according to the 2024 CLSI guidance using iron-depleted cation-adjusted Mueller-Hinton broth (ID-CAMHB) prepared from Chelex resin and BD-BBL Mueller-Hinton broth powder. MICs of FDC in combination with 4 µg/mL of XER were also determined using ID-CAMHB.

**Methods:**

A total of 19 isolates (6 Enterobacterales (ENT), 6 *Pseudomonas aeruginosa* (PA) and 7 *Acinetobacter baumannii* complex (ABC)) with FDC MIC values ranging from 0.5 to >32 µg/mL were used. The β-lactamase profile for each isolate was determined by PCR or whole genome sequencing. Mice were infected in the thigh and dosed subcutaneously with a humanized dosage regimen of FDC, 100 mg/kg q2h, with or without XER 20 mg/kg q2h, over 24 hours (both dosage regimens achieved 100% and 81.7% free time above MIC against 4 and 8 mg/L, respectively), starting 2 hours after inoculation. Change in viable bacterial cell counts in the thighs were determined from start to end of treatment.

**Results:**

In the 6 ENT strains, treatment with FDC resulted in no bacterial killing for 3 isolates with MIC values >4 µg/mL, the addition of XER resulted in bacterial killing in these isolates (Table). In the 7 ABC strains, treatment with FDC resulted in no bacterial killing for isolates with MICs >32 µg/mL, the addition of XER was able to restore the activity of FDC. FDC/XER was not effective against 1 ABC isolated with a FDC/XER MIC of 16 µg/mL. In the 6 PA isolates, treatment with FDC resulted in bacterial killing against 5 of the 6 isolates. The addition of XER resulted in bacterial killing against all 6 PA isolates.

**Conclusion:**

The addition of XER significantly increased the efficacy of FDC in a murine thigh infection model against ENT and ACB.

**Disclosures:**

Yoshinori Yamano, PhD, Shionogi HQ: Employee

